# Initial Unilateral Exposure to Deep Brain Stimulation in Treatment-Resistant Depression Patients Alters Spectral Power in the Subcallosal Cingulate

**DOI:** 10.3389/fncom.2018.00043

**Published:** 2018-06-12

**Authors:** Otis Smart, Ki S. Choi, Patricio Riva-Posse, Vineet Tiruvadi, Justin Rajendra, Allison C. Waters, Andrea L. Crowell, Johnathan Edwards, Robert E. Gross, Helen S. Mayberg

**Affiliations:** ^1^Department of Neurosurgery, Emory University School of Medicine, Atlanta, GA, United States; ^2^Department of Psychiatry and Behavioral Sciences, Emory University School of Medicine, Atlanta, GA, United States; ^3^Department of Biomedical Engineering, Georgia Institute of Technology, Atlanta, GA, United States

**Keywords:** depression, LFP, SCC, DBS, power spectra, tractography, VTA/VTR, intraoperative

## Abstract

**Background:** High-frequency Deep Brain Stimulation (DBS) of the subcallosal cingulate (SCC) region is an emerging strategy for treatment-resistant depression (TRD). This study examined changes in SCC local field potentials (LFPs). The LFPs were recorded from the DBS leads following transient, unilateral stimulation at the neuroimaging-defined optimal electrode contact. The goal was identifying a putative electrophysiological measure of target engagement during implantation.

**Methods:** Fourteen consecutive patients underwent bilateral SCC DBS lead implantation. LFP recordings were collected from all electrodes during randomized testing of stimulation on each DBS contact (eight total). Analyses evaluated changes in spectral power before and after 3 min of unilateral stimulation at the contacts that later facilitated antidepressant response, as a potential biomarker of optimal contact selection in each hemisphere.

**Results:** Lateralized and asymmetric power spectral density changes were detected in the SCC with acute unilateral SCC stimulation at those contacts subsequently selected for chronic, therapeutic stimulation. Left stimulation induced broadband ipsilateral decreases in theta, alpha, beta and gamma bands. Right stimulation effects were restricted to ipsilateral beta and gamma decreases. These asymmetric effects contrasted with identical white matter stimulation maps used in each hemisphere. More variable ipsilateral decreases were seen with stimulation at the adjacent “suboptimal” contacts, but changes were not statistically different from the “optimal” contact in either hemisphere despite obvious differences in impacted white matter bundles. Change in theta power was, however, most robust and specific with left-sided optimal stimulation, which suggested a putative functional biomarker on the left with no such specificity inferred on the right.

**Conclusion:** Hemisphere-specific oscillatory changes can be detected from the DBS lead with acute intraoperative testing at contacts that later engender antidepressant effects. Our approach defined potential target engagement signals for further investigation, particularly left-sided theta decreases following initial exposure to stimulation. More refined models combining tractography, bilateral SCC LFP, and cortical recordings may further improve the precision and specificity of these putative biomarkers. It may also optimize and standardize the lead implantation procedure and provide input signals for next generation closed-loop therapy and/or monitoring technologies for TRD.

## Introduction

High-frequency, bilateral Deep Brain Stimulation (DBS) of the subcallosal cingulate (SCC) region is an emerging strategy for treatment-resistant depression (TRD). Six-month response rates across experimental trials have been variable (rates of 22–66% reported), but sustained long-term antidepressant effects with continuous stimulation appear more consistent (Mayberg et al., 2005; Lozano et al., 2008, 2012; Guinjoan et al., 2010; Kennedy et al., 2011; Holtzheimer et al., 2012, 2017; Puigdemont et al., 2012, 2015; Merkl et al., 2013; Ramasubbu et al., 2013; Crowell et al., 2015).

As with other clinical applications of DBS, the precise localization of delivered stimulation is an essentiality. With SCC DBS for TRD, recent refinements of DBS surgical targeting methods using diffusion tractography demonstrate that stimulation of 4 white matter bundles—the cingulum bundle (CB), uncinate fasciculus (UF), forceps minor (FM) and fronto-striatal fibers (F-St)—converging at the anatomically defined SCC DBS target in each hemisphere is necessary for positive outcomes (Riva-Posse et al., 2014, 2017). The implementation of an optimized connectomic approach resulted in a response rate of 72.7%, up from a 41% using standard anatomical landmarks in the same protocol (Riva-Posse et al., 2017).

Confirmation of correct placement in each hemisphere while in the operating room is an important next step. Toward this goal, controlled experiments demonstrate that immediate and stereotypic self-reported behavioral changes can be elicited with short exposures to unilateral stimulation using therapeutic DBS parameters (Choi et al., 2015). Such observations provide important foundation to examine electrophysiological changes with unilateral stimulation as a more quantitative biomarker strategy to validate effective surgical targeting in each hemisphere.

That stated, direct recordings from this DBS target is a difficult technique, as it is predominantly in the white matter. Several reports demonstrate, however, that local field potentials (LFPs) can be detected in this region during insertion of the lead (Neumann et al., 2014; Clark et al., 2016) and in response to emotional tasks (Merkl et al., 2016). These preliminary studies utilize different recording techniques and different depression rating scales, but nonetheless suggest that local patterns of oscillatory activity may have potential clinical relevance: increased SCC region alpha power and decreased SCC region beta power both show significant correlations with baseline depression symptom severity, respectively. Related studies have explored basal SCC activity as a biomarker of MDD treatment response to non-DBS interventions, notably medication (Pizzagalli et al., 2004), medication and psychotherapy (Konarski et al., 2009; McGrath et al., 2014), sleep deprivation (Wu et al., 1992) and cingulotomy (Dougherty et al., 2003). These studies have utilized resting state measures of regional glucose metabolism as well as source localization approaches derived from scalp EEG (e.g., Pizzagalli et al., 2001, 2004), setting the foundation for the proposed new experiments. Changes in SCC activity with treatment have also been consistently demonstrated with decreases seen with DBS (Mayberg et al., 2005; Lozano et al., 2008) as well as with medication (Mayberg et al., 2000; Kennedy et al., 2007). No studies to date, have examined acute, intraoperative stimulation effects following first exposure to SCC DBS.

In this study, we examined changes in LFPs within the SCC following transient, unilateral SCC stimulation at DBS contacts known to elicit both acute intraoperative behavioral effects and facilitate sustained chronic antidepressant response with bilateral stimulation (Holtzheimer et al., 2012; Choi et al., 2015; Riva-Posse et al., 2017). We capitalized on the opportunity to record these brain signals as part of the routine behavioral testing performed during DBS lead implantation surgery. The local field potential (LFP) signals were recorded following the first exposure to high frequency stimulation (HFS) at the therapeutically effective contacts to identify a putative electrophysiological signal of optimal placement and target engagement in each hemisphere. Analyses focused on two main research questions: (1) are there differential spectral frequency band changes with left and right unilateral stimulation at the effective contacts; (2) are identified changes specific to the effective contact?

## Materials and methods

### Patients

Fourteen consecutive TRD patients were enrolled in ongoing experimental trials of SCC DBS (gender: 12 women and 2 men; age: 30–65 years) (IDE G060028, clinicaltrials.gov NCT00367003; IDE: G130107, NCT01984710). Informed consent was obtained from all patients in compliance with Emory Institutional Review Board policies.

### Surgery

**Figure 1:**
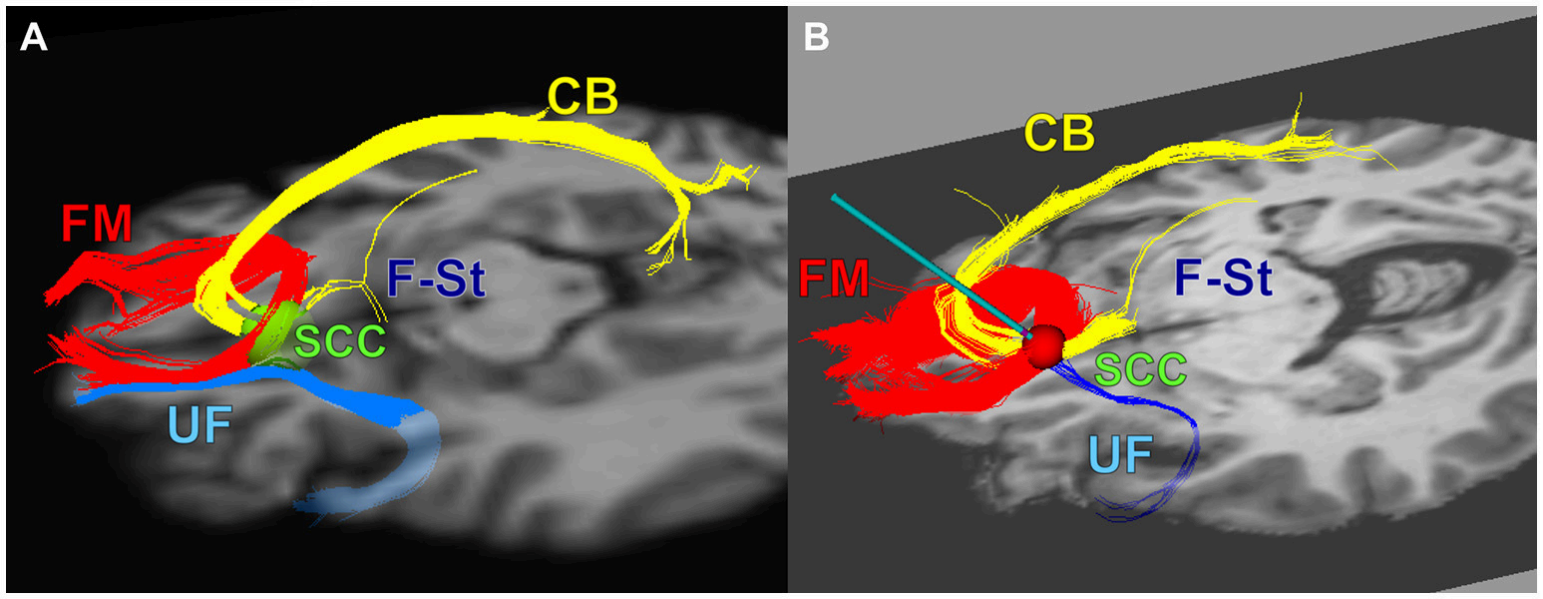
The combined tractography and anatomical images guided a connectomic surgical procedure to implant the subcallosal cingulate DBS leads. **(A)** connectomic blueprint used for structural connectivity based target selection (intersection of forceps minor, uncinate fasciculus, cingulum bundle, and fronto-striatal fibers), **(B)** a representative deterministic tractography target selection map from one patient: optimal target location within SCC region with modeled stimulation impacting necessary fiber bundles for effective SCC DBS. FM, Forceps Minor; UF, Uncinate Fasciculus; CB, Cingulum Bundle; F-St, fronto-striatal fibers. **(A)** adapted from Figure 4 in Riva-Posse et al. (2014).

Patients were implanted bilaterally with one DBS lead per hemisphere (4 electrode contacts per lead) as previously published (Riva-Posse et al., 2017). The frame-based stereotactic surgical procedure combined deterministic tractography and high-resolution T1-Weighted structural MRI to identify the desired target at the intersection of four white-matter bundles in the SCC region (Riva-Posse et al., 2017) using a standard intraoperative planning workstation (StealthStation, Medtronic, Louisville, CO) (Figure 1).

Twelve patients were implanted with the Libra DBS system (St Jude Medical, Plano, TX). These electrodes have the following specifications: diameter: 1.4 mm, length: 1.5 mm [electrodes 2–4], length: 3 mm [electrode 1], center-to-center: 3 mm, inter-electrode space: 1.5 mm. The remaining two patients received Activa PC+S 3387 leads (electrodes with diameter:1.27 mm, length: 1.5 mm, center-to-center: 3 mm, inter-electrode space 1.5 mm) (Medtronic, Minneapolis, MN). A nomenclature of L1-L4 and R1-R4 was used for the four ventral-to-dorsal electrodes within the left (L) and right (R) hemispheres respectively, for both types of leads.

### Intraoperative behavioral testing of tractography defined DBS targets

**Figure 2:**
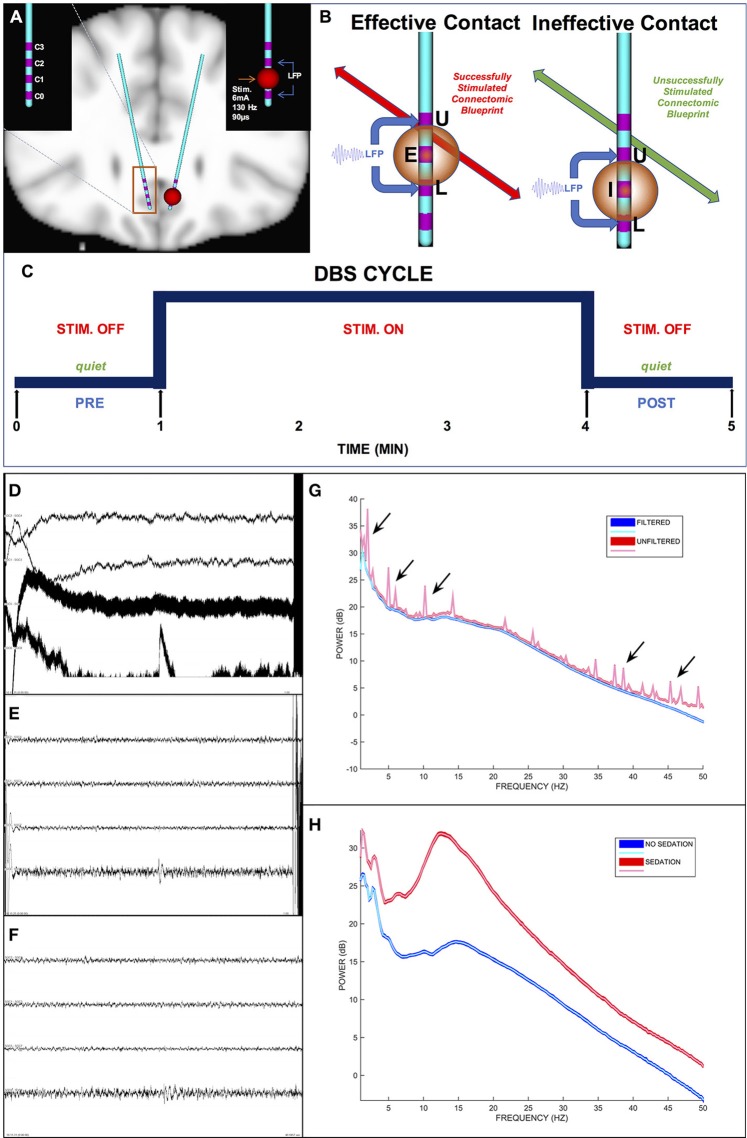
Intraoperative DBS-Evoked behavioral testing and stimulation protocol. **(A)** The location of DBS leads within the subcallosal region. **(B)** Tractography-based verification of the defined effective and ineffective targets with a differential signal recording. **(C)** Intraoperative stimulation protocol. **(D)** A 1-min epoch of unfiltered differential signals. **(E)** The same epoch in **(D)** but filtered differential signals including artifacts (i.e., high amplitude deflections at the beginning and end of the epoch). **(F)** The same epoch in **(E)** after filtration and artifact removal (notice the clipped time interval). **(G)** Power spectra of the unfiltered (red curves) and filtered (blue curves) epochs. The arrows indicate representative spectral noise in the unfiltered signal not present in the filtered signal. **(H)** Power spectra of filtered signals for 1-min epochs during sedation (red curves) and no sedation (blue curves). In **(D–F)**, the differential signals are L1–L3, L2–L4, R1–R3, and R2–R4 respectively. E, Effective contact; I, Ineffective contact; U, Upper VTR; L, Lower VTR.

DBS lead implantation was conducted with patients under light propofol anesthesia without intubation. Once both leads were implanted and secured, anesthesia was discontinued and patients were awakened for behavioral assessments. Patients were fully alert for a minimum of 60 min prior to initiation of any behavioral testing or electrophysiological recordings to minimize contamination by residual propofol effects (Figure 2H, cf. Purdon et al., 2013).

Each patient received active monopolar unilateral electrical HFS at each of the 8 contacts (Figures 2A,B) and at 4 sham contacts (no stimulation) to confirm the contact in each hemisphere with the most robust behavioral response. We termed each HFS trial (Figure 2C) a “cycle,” which consisted of a 1 min pre-DBS interval (i.e., “DBS PRE”), 3 min of continuous DBS on a single particular contact (i.e., “DBS”), and a 1 min post-DBS interval (i.e., “DBS POST”). The HFS was applied by an external neurostimulator with a surface skin electrode as ground to deliver electrical stimulation at 6 mA amplitude, 130 Hz frequency, and 90 μs pulse width per phase.

The patients and examiner were all blinded to all DBS cycle conditions. Patients were instructed to remain completely quiet throughout each 5 min cycle except for a fixed-time interval (i.e., the first 1 min after initiation of stimulation) to self-report changes in their moods, feelings, thoughts, or sensations to a testing examiner. An analysis of these behavioral reports was conducted in a separate study (Choi et al., 2015). The patients did not answer any self-report questions in the 1 min post-stim intervals or the 1 min pre-stim intervals to prevent confounds in LFP records and analytics.

### Electrophysiology recordings

For each patient, a continuous LFP recording was simultaneously collected from all eight DBS electrodes throughout the entire intraoperative DBS-evoked behavior testing session. Since the clinical outcomes were not determined until 6 months after the intraoperative procedure, we collected data from all electrodes.

The continuous LFP records were collected at 1,000 Hz (samples per second) throughout the entire intraoperative behavioral test with either a TDT RZ Processor with PZ2 Amplifier (Tucker-Davis Technologies, Alachua, FL, USA) or an XLTEK EMU40EX processor with amplifier (Natus Excel-Tech Corporation, Oakville, Ontario, Canada). Both systems used a common reference and common ground acquisition setup. Data digitization was 16 bits per sample and either 1,024, 1,525, or 2,034 Hz. For offline LFP analytics, the data were exported to EDF+ files (Kemp and Olivan, 2003) then downsampled to 128 Hz.

### Clinical follow-up and determination of the effective response contacts for retrospective analyses of acute intraoperative DBS effects

Chronic bilateral HFS was initiated 4 weeks after surgery. Stimulation was delivered through a single contact per lead using standardized DBS parameters (frequency: 130 Hz, pulse width: 90 μsec, current: 6–8 mA for St. Jude Medical devices, voltage: 3.5–5 V for Medtronic devices). Contact selection for chronic stimulation was based on a combination of factors, specifically the proximity of the lead contact to the ideal tractography target, with confirmatory verification of a intraoperative behavioral response (Riva-Posse et al., 2017). These targets were referred to as the “effective” contacts for the analyses described below. Clinical response to DBS was defined as a 50% decrease in the 17-item Hamilton Depression Rating Scale (HDRS) (Hamilton, 1960) at 6 months.

### Contact selection criteria

Once clinical determination of the DBS contacts with significant antidepressant responses were defined, a retrospective analysis of the intraoperative LFP changes with HFS was performed. The two contacts used for chronic stimulation (i.e., “Effective”) and the immediately adjacent control contacts (i.e., “Ineffective”) were selected (Figure 2B).

### LFP signal processing

Each pair of “DBS PRE” and “DBS POST” HFS epochs were extracted from the LFP recordings and input into a custom semi-automated algorithm that computed the HFS effects as bandlimited relative power changes (i.e., “DBS POST” vs. “DBS PRE”). Subsequently, the bandlimited power change values and their corresponding labels (e.g., stim: left effective, signal: left, band: alpha) were input into an additional algorithm that computed inferential statistics (section Statistical Analyses).

#### Artifact rejection

Only artifact-free LFP signals (Figure 2F) were analyzed. The timestamps (i.e., start time and end time) of the LFP artifacts (e.g., electrical, muscle, motion) within each DBS cycle were manually identified and annotated using EDFbrowser software (http://www.teuniz.net/edfbrowser/) to visual review of the signals. All timestamps of the annotated artifacts were stored in a file as input data to a custom semi-automated algorithm (see section Power Spectral Analyses) that performed power spectral analyses after ignoring (discarded not blanked) all LFP signal values inclusively within the input timestamps.

Signal amplitudes greater than ~100 μV in magnitude and greater than ~250 ms in duration indicated an artifact (Figure 2E). The reliability of the signal cleaning process was ensured by contrasting the signal power spectra before and after pruning of artifacts (Figure 2G).

Across all 896 total epochs (i.e., 14 patients, 8 signals, 4 cycles, and 2 pre-stim and post-stim intervals), the visual review identified 542 artifacts (duration mean: 8.60 ± 0.56 s; duration std: 12.98) and their removal resulted in ~8.68% total lost data.

#### Definition of a differential signal

In addition to the above artifact rejection approach, we used differential LFP signals (Figure 2E) rather than referential LFP signals (Figure 2D) to eliminate the effects caused by variations in the system reference electrode as well as common-mode noise in any electrode. Also, electrical HFS artifacts saturated the amplifiers of the LFP recording systems, thereby precluding the use of referential LFP signals for suitable analytics.

Each differential signal was derived from the two referential signals surrounding each electrode of interest (Figure 2B). For instance, if L2 and R3 were the clinically determined effective response contacts (see above section) for a patient, then L1–L3 and R2–R4 respectively were the derived differential signals for ensuing analytics (Figure 2F). This technical limitation restricted analyses to the middle two contacts per lead, defined above as either Effective or Ineffective.

#### Power spectral analyses

Custom MATLAB R2015a (The MathWorks Inc., Natick, MA, USA) scripts were created then used to compute and tabulate artifact-free bandlimited power change values per patient in the following manner using both the LFP recordings and the file of annotated artifact timestamps as input data.

The eight referential LFP signals of each DBS cycle were bandpass filtered (1–50 Hz) with zero-phase delay (filtfilt.m), decimated to 128 Hz, pruned of artifacts [i.e., discard of signal amplitudes with time points that coincide the annotated timestamps of artifacts (see section Artifact Rejection)], modified to become four differential LFP signals, and divided into the 1 min DBS PRE and 1 min DBS POST epochs. The LFP power spectrogram (i.e., time-frequency-power matrix) was calculated per epoch per differential signal using the CWT (continuous wavelet transform) technique (i.e., cwt.m) with a complex-morlet wavelet and 200 frequency points linearly spaced between 1 and 50 Hz (i.e., 0.25 Hz frequency resolution). Each pair of “DBS PRE” and “DBS POST” spectrograms were converted to a single power spectrum (i.e., frequency vs. relative power change) by applying a bootstrap technique (i.e., bootci.m) with a 95% CI (confidence interval) and this formula (Equation 1) as the estimator (bootstrap statistic) per frequency point across all its time points.

(1) $$\begin{array}{l} relativechange\left(v_{DBSPOST},v_{DBSPRE}\right)\\ \;=\frac{\left(median\left(v_{DBSPOST}\right)-median\left(v_{DBSPRE}\right)\right)}{median\left(v_{DBSPRE}\right)} \end{array}$$

In this equation, *v*_*DBSPOST*_ was the CWT magnitudes for the DBS POST epoch and *v*_*DBSPRE*_ was the CWT magnitudes for the DBS PRE epoch. Thus, for each frequency point, the algorithm calculated the CI minimum, the CI maximum, and the CI midpoint (which was derived from the maximum and minimum). Both the bootstrap and the median function within the relative change formula avoided any parametric presumptions on the distributions of power values and provided robustness in power measures. The relative change statistic quantified the acute effects of DBS and controlled for potential changes over time in the electrophysiology measures throughout the intraoperative testing.

Finally, the bandlimited power change measures were computed as the mean of all CI midpoints within each of the following frequency band ranges: delta (δ: 1–4 Hz), theta (θ: 4–8 Hz), alpha (α: 8–15 Hz), beta (β: 15–30 Hz), and gamma (γ: 30–50 Hz). Hence, this process overall reduced the original 200 frequency points to 5 frequency bins and condensed the 7,680 (128 × 60) maximum time points per epoch to 1 single time bin.

The bandlimited power change measures across all combinations of patients, DBS cycles, and LFP differential signals formed multiple statistical samples for hypothesis-testing analytics. The sample sizes varied across the defined contrasts since some patients had missing values due to the inability to compute a differential signal (i.e., an Effective electrode of L4 or/and R4, which only had one adjacent electrode rather than the needed two adjacent electrodes).

### Quantification of the volume and location of the stimulated region

We performed tractography-based verification of the defined Effective (treatment) and Ineffective (control) targets. Using previously published methods (Riva-Posse et al., 2014, 2017; Choi et al., 2015; Noecker et al., 2017), the patient-specific DBS volume of tissue activated (VTA) was generated by the electrical DBS field model method (Chaturvedi et al., 2013). The VTA was used as a seed to generate a probabilistic tractography map of impact WM bundles using the post-operative X-ray Computed Tomography (CT) merged with the preoperative high-resolution structural MRI and diffusion scans. A mean response connectivity map of Effective and Ineffective contacts was calculated by averaging the probability connectivity maps across patients. These maps were used to interpret differences in impacted tracts between the Effective and Ineffective contacts as well as potential differences between left and right hemisphere LFP findings.

In addition to a VTA, a spherical volume of tissue recorded (VTR) was created on the center of the corresponding LFP recording contacts (radius = 7.5 mm), which were one contact above (Upper VTR) and one contact below (Lower VTR) the active stimulation contact, to compare the differences in the current sources between left and right effective/ineffective contacts (relative amount of gray and white matter) (Lindén et al., 2011; Lempka and McIntyre, 2013). LFPs are believed to originate primarily from the electric potentials generated by transmembrane currents in the dendrites and soma of gray matter, and the current sources that contribute to these recordings are thought to extend out to several millimeters when recording with macroscopic electrodes. We used the FSL tissue segmentation toolbox (FAST, FMRIB Software Library, http://www.fmrib.ox.ac.uk/fsl; Jenkinson et al., 2012) to segment gray matter, white matter, and cerebrospinal fluid. The intersection volumes of the VTRs with the binary masks from tissue segmentation were calculated. Moreover, the anatomical structures contained within the Effective and Ineffective VTRs on the left and right were defined using the Freesurfer (Fischl, 2012) cortical parcellation (Destrieux Atlas, Destrieux et al., 2010) tool to further characterize regional contributors to recordings in each hemisphere. The volume of the four cortical and subcortical parcellated gray matter regions were compared among upper and lower VTRs in the left and right hemispheres (**Figure 5**. ACC: Anterior Cingulate Cortex, Sub. OFC: Suborbital Frontal Cortex, SCC: Subcallosal Cingulate, nAc/Caudate: Nucleus Accumbens/Caudate).

### Statistical analyses

Statistics were computed using MATLAB R2015a (The MathWorks Inc., Natick, MA, USA) software. Visuals of the descriptive statistics were generated using R statistical packages (The R Project for Statistical Computing, www.r-project.org). Statistics were restricted only to the 6-month responders.

Unequal-tail one-sample Kolmogorov-Smirnov tests confirmed non-gaussianity of the power spectra values. Exact two-tailed Wilcoxon-Mann-Whitney rank-sum tests were used to identify any difference from zero in the power spectra measures for each the effective and ineffective contacts separately. Exact two-tailed Wilcoxon signed-rank tests for were used to compare power spectra measures of effective vs. ineffective response contacts paired.

The Bonferroni correction for the multiple comparisons (5 bands per person per DBS target per hemisphere) was used to adjust a *p* < 0.05 significance level; thus the new significance threshold was *p* < 0.05/5 = 0.01. Hedges' g was computed as an effect size statistic. When applicable, exact hypothesis-testing methods were used given the small sample size of this study.

## Results

### Patient response characteristics

Eleven of the fourteen patients met criteria for antidepressant response by 6 months with stimulation of the effective contacts defined above (Table 1). Of the three 6-month non-responders, 1 responded after the 6-month study endpoint but without a contact change (Patient 2), 1 responded after a contact switch in the left hemisphere (Patient 7), and 1 remained a non-responder (Patient 6).

**Table 1:** Patient characteristics.

**Patient**	**Baseline (HDRS17)**	6 months^*^ (HDRS17)	Time to stable response (weeks)^**^
1	25.5	12	26
2	24.25	13	40
3	27.5	6	8
4	23.75	9	25
5	22	6	24
6	22.75	16	NA
7	20.25	17	74
8	26.25	12	22
9	20	7	4
10	25.25	6	5
11	21	6	4
12	21.5	9	4
13	29.25	3	3
14	23.25	8	19
Mean (sd)	23.8 (2.8)	9.6 (4.5)	19.9 (20)

* *Italicized–Responders at 6 Months (50% decrease in HDRS17 from Baseline)*.

** *3 consecutive weeks with a 50% decrease in the HDRS17*.

### DBS-evoked power changes in the defined effective response contacts

**Figure 3:**
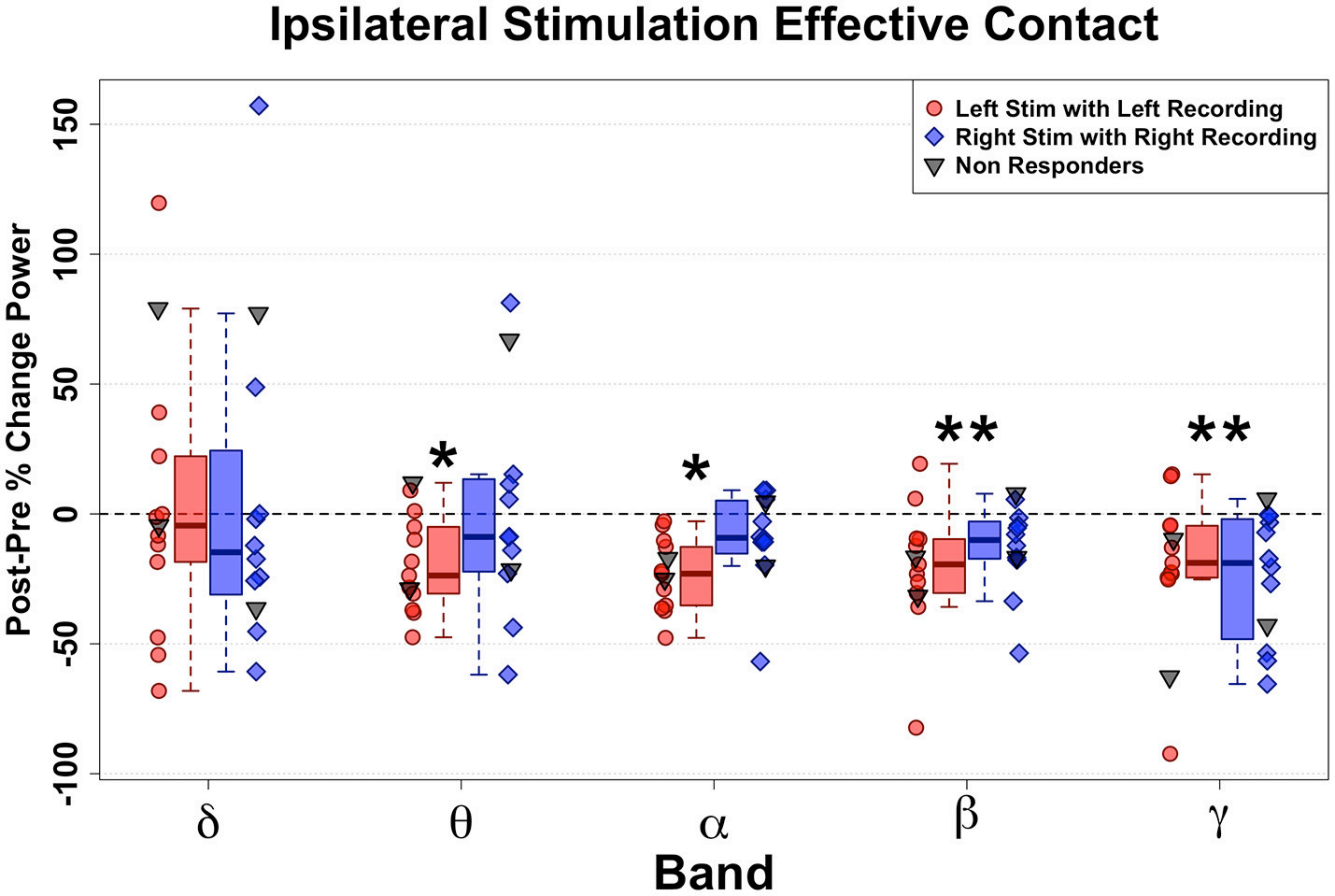
Ipsilateral band-delimited power changes with Effective DBS in Responders (*n* = 11). Decreased spectral power ipsilateral to site of HFS is demonstrated. Differential patterns are seen following Left-sided and Right-sided DBS. Left-sided HFS with Left SCC recording in Red. Right-sided HFS with Right SCC recordings in Blue. Grouped patient values are represented by the box plots (Responders only). Individual patient values are represented by dots (with non-responders shown in gray). Significant decreases were induced by Left effective stimulation in Theta, alpha, beta and gamma band. More limited decreases were induced by right effective stimulation in beta and gamma bands. Statistically significant changes are marked with an asterisk.

Transient unilateral intraoperative stimulation at effective left and right DBS contacts both evoked significant changes in respective ipsilateral SCC LFP, but with different patterns for left-sided and right-sided stimulation. Overall, HFS in the effective DBS target resulted in decreased spectral power (Figure 3). Left-sided DBS evoked ipsilateral broadband reductions in left SCC activity spanning theta (*n* = 11, *W* = 88, *p* = 0.005, *g* = −1.58), alpha (*n* = 11, *W* = 66, *p* < 0.001, *g* = −2.17), beta (*n* = 11, *W* = 88, *p* = 0.005, *g* = −1.06), and gamma (*n* = 11, *W* = 88, *p* = 0.005, *g* = −0.85) bands (Figure 3, in red) with the alpha band having the largest decrease, followed by theta. In contrast, right-sided DBS evoked more restricted but significant ipsilateral spectral reductions in right SCC beta (*n* = 10, *W* = 65, *p* < 0.001, *g* = −1.14) and gamma (*n* = 10, *W* = 55, *p* < 0.001, *g* = −1.37) bands (Figure 3, in blue).

Since there were only three non-responders for this study, group-mean analyses had no practical value. Therefore, individual non-responders were plotted (Figure 3, gray dots) in juxtaposition with the responders. Interestingly, the two non-responders at 6-months who experienced response after 6 months both showed a similar acute change pattern to the Responder group (Figure 3, blue and red dots): Patient 2 (not shown), who went on to a full response without change in stimulation contact; and Patient 7, who responded after changing the left-hemisphere stimulation contact. In contrast, the other non-responder (Patient 6) remained a long-term non-responder and showed the most deviant pattern from the rest of the group.

### DBS-evoked power changes in ineffective vs. effective contacts

**Figure 4:**
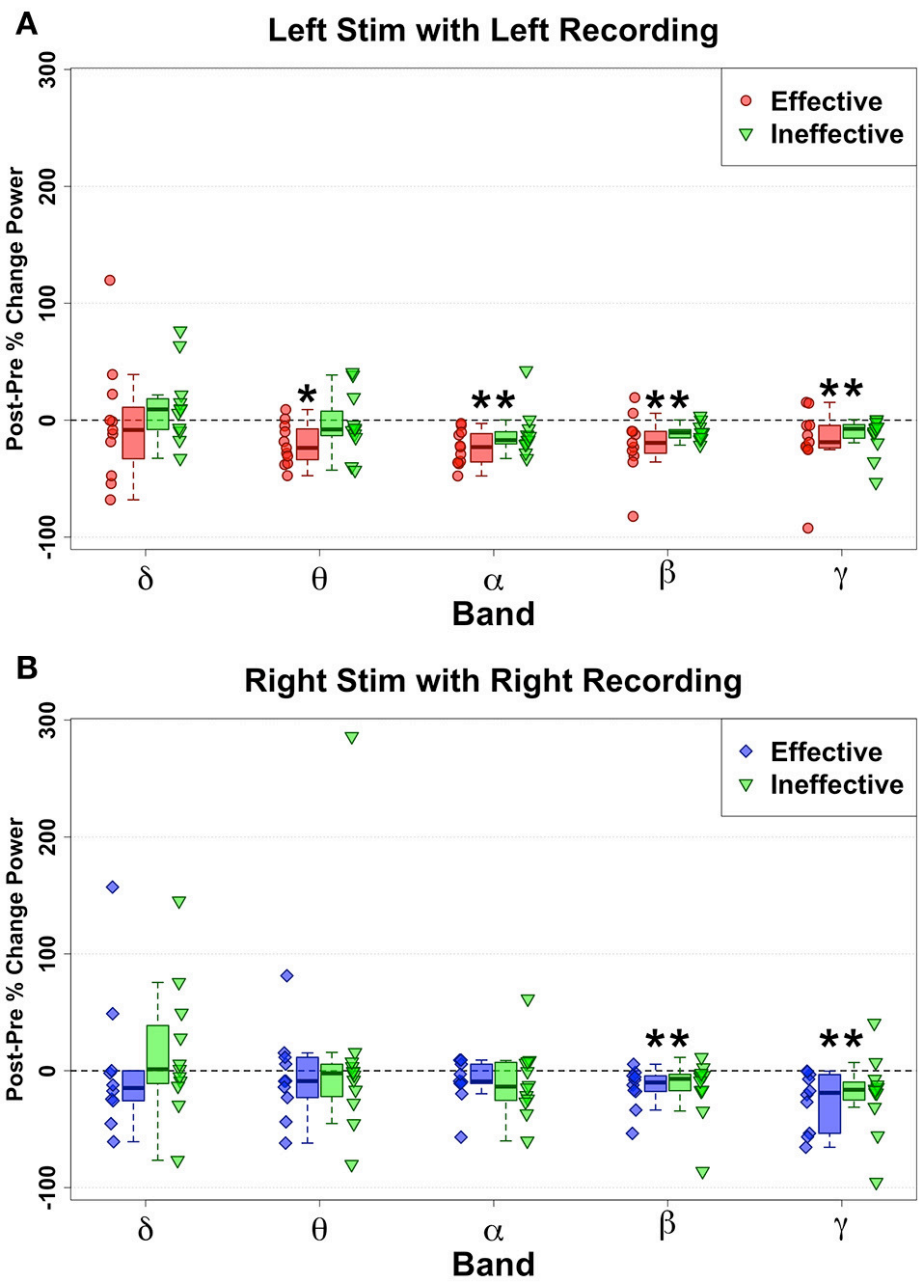
Ipsilateral change pattern contrasting HFS delivered to effective Left (red) and ineffective left (green) contacts **(A)** and Effective Right (Blue) and ineffective right (green) contacts **(B)**. Decreased spectral power ipsilateral to site of HFS is demonstrated with ineffective contacts. But no significant differences were identified via direct comparison of effective and ineffective contacts, although Left Theta decreases show a trend (*p* = 0.043) to differentiate effective from ineffective contacts. Statistically significant changes are marked with an asterisk.

Similar to changes induced with HFS to effective contacts, HFS to ineffective contacts also elicited decreases in power, but with a different pattern across frequency bands. Significant changes with stimulation of left-sided ineffective contacts included alpha (*n* = 11, *W* = 88, *p* = 0.005, *g* = −0.78), beta (*n* = 11, *W* = 88, *p* = 0.005, *g* = −1.90), and gamma (*n* = 11, *W* = 88, *p* = 0.005, *g* = −1.11) bandwidths (Figure 4A). Notably, no significant changes were seen in the theta (*n* = 11, *W* = 99, *p* = 0.057, *g* = −0.171) band, where effective stimulation showed a robust and consistent decrease.

However, direct comparison of changes between Left Effective and Left Ineffective HFS showed no significant differences for any band: LSCC delta (*n* = 11, *W* = 19, *p* = 0.240, *g* = −0.34), theta (*n* = 11, *W* = 11, *p* = 0.053, *g* = −0.93), alpha (*n* = 11, *W* = 13, *p* = 0.083, *g* = −0.78), beta (*n* = 11, *W* = 24, *p* = 0.465, *g* = −0.507), and gamma (*n* = 11, *W* = 30, *p* = 0.831, *g* = −0.166). While not statistically significant, the distribution of changes in theta with ineffective stimulation showed a bimodal distribution not seen in other bands.

Right-hemisphere HFS of ineffective contacts, like at effective contacts, evoked ipsilateral narrowband reductions in SCC activity (Figure 4B) in the beta (*n* = 11, *W* = 77, *p* < 0.001, *g* = −0.95) and gamma bands (*n* = 11, *W* = 88, p < 0.006, *g* = −0.80). As observed with left-sided HFS, neither beta (*n* = 10, *W* = 34, *p* = 0.557, *g* = 0.99) nor gamma bands (*n* = 10, *W* = 36, *p* = 0.432, *g* = −0.07) distinguished effective from ineffective HFS effects. Likewise, there were no significant changes with ineffective HFS or any significant Effective-Ineffective change pattern differences in the remaining bandwidths: RSCC delta (*n* = 10, *W* = 19, *p* = 0.432, *g* = −0.26), theta (*n* = 10, *W* = 45, *p* = 0.084, *g* = 0.47), and alpha (*n* = 10, *W* = 40, *p* = 0.232, *g* = 0.630).

### Evaluating similarities and differences in tractography and VTR maps of effective and ineffective contacts

**Figure 5:**
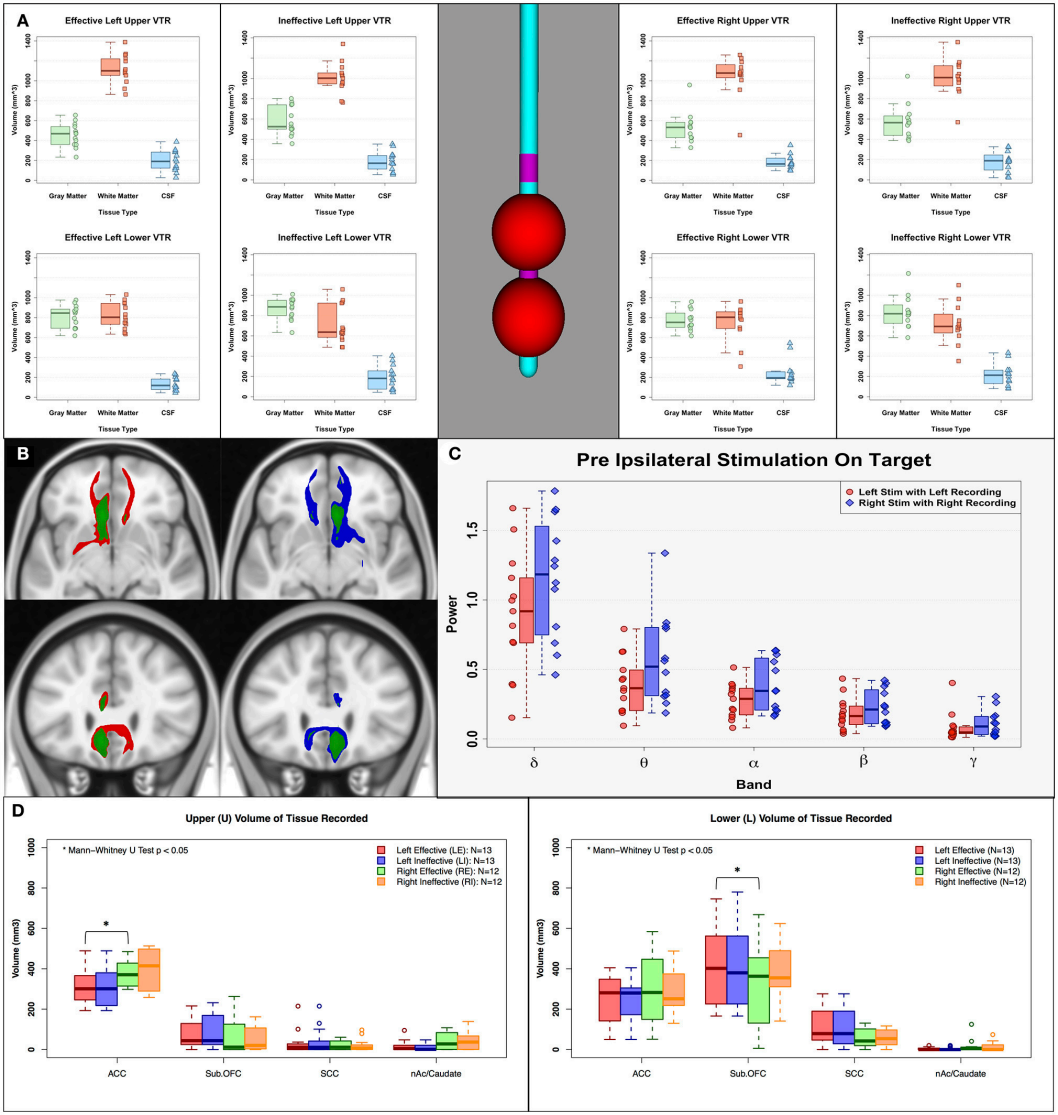
Sources of asymmetry. **(A)** Estimated volume of tissue recorded (VTR: 7.5 mm radius sphere) on the center of the corresponding LFP recording contacts, **(B)** Left mean response connectivity map of Effective (Red) and Ineffective (Green) contacts, and Right mean response connectivity map of Effective (Blue) and Ineffective (Green) contacts, **(C)** Pre-stimulation power of left and right effective contacts. **(D)** Estimated anatomical current sources within VTRs using FreeSurfer cortical parcellation. There are no clear sources of asymmetry between left and right hemisphere in pre-stimulation power, amount of gray/white/CSF within volume of tissue recorded, or impacted white matter bundles. However, there are clear differences in anatomical current sources within the VTRs; left received less input from the ACC compared to the right, and right received less input from the suborbital frontal cortex. ACC, Anterior Cingulate Cortex; Sub. OFC, Suborbital Frontal Cortex; SCC, Subcallosal Cingulate; nAc/Caudate, Nucleus Accumbens/Caudate.

In contrast to the findings comparing Effective to Ineffective LFP changes, Effective vs. Ineffective tractography maps showed distinct and consistent differences. Tractography of the mean Effective vs. Ineffective maps both showed comparable involvement of the cingulate bundle but with obvious differences in the impact on the forceps minor (Figure 5B). Ineffective contacts have consistently fewer streamlines within the FM than effective contacts. Failure to stimulate the FM was the most consistent finding in previous studies defining the combination of white matter bundles necessary for clinically significant DBS response (Riva-Posse et al., 2014, 2017).

### Evaluating sources of asymmetric responses with left and right SCC DBS

The asymmetric ipsilateral effects of effective stimulation to the left and right SCC were examined using assessments of differences in the respective structural connectivity maps and volumes of tissue recorded (VTR). The left-right differences cannot be attributed to any sampling differences of the SCC region due to placement of the DBS leads or the composition of gray matter, white matter and CSF within the VTR (Figure 5A). These ratios were comparable for right and left contacts across the response groups. Similarly, the left-right change pattern differences with HFS were not related to a difference in the tractography maps of white matter fibers impacted by acute stimulation, which were identical for the left-sided and right-sided contacts (Figure 5B, red and blue maps, respectively). Basal asymmetry in pre-stimulation SCC power was also not a factor (Figure 5C). Although the left and right VTRs had a similar composition of gray matter within VTRs, there were anatomical differences in the current sources that contributed to these recordings. Left VTRs received less from the ACC compared to right (Figure 5D-left panel; Mann-Whitney paired *U*-test *p* < 0.05). In contrast, right VTRs received less input from the suborbital frontal cortex than left (Figure 5D-right panel).

## Discussion

This study identified acute changes in SCC LFP recordings during intraoperative testing of unilateral HFS at tractography-defined contacts that later led to long-term antidepressant response. These changes were observed ipsilateral to the side of stimulation and, despite similar delivery of HFS to each hemisphere, the oscillatory response was asymmetric: left-sided stimulation evoked broadband effects, and right-sided stimulation effects were restricted to beta and gamma bands. While these observations characterized first effects of stimulation at the tractography-defined “ideal” location, these changes proved non-specific, as similar power decreases were seen with HFS at the adjacent “ineffective” control contacts. However, theta power decreases with left effective HFS were both more robust and more consistent than left “ineffective” HFS, suggesting a potential optimization biomarker for further study. Similarities in both magnitude and variance of LFP changes with “effective” and “ineffective” right-sided stimulation further suggest that precision of right sided contact selection may be less critical than on the left.

These lateralized decreases (i.e., broadband changes on the left, restricted decreases on the right) mirror previous PET studies of DBS effects that show greater left than right SCC blood flow decreases (Mayberg et al., 2005). Lateralized behavioral responses to SCC stimulation have also been demonstrated in this same cohort, with left-sided stimulation generating consistently more robust behavioral effects (Choi et al., 2015). Asymmetric cortical phase-coherence in chronically treated DBS patients has been reported (Quraan et al., 2014), suggesting that these lateralized acute SCC effects may have chronic correlates.

Tissue differences, including specific brain regions or cell types sampled, or more nuanced differences in the proportion of gray and white matter within the VTR may contribute to the apparent differential signals recorded from the right and left hemispheres (see section Quantification of the Volume and Location of the Stimulated Region). In support of this hypothesis, a *post-hoc* analysis of the anatomical parcellation of the VTR demonstrated that, in this cohort, the left Effective VTR samples less ACC than right, while the right Effective VTR samples less suborbital frontal gray matter than the left VTR. While further parcellation of the LFP signal is not a possibility given the resolution of the recording capabilities of the DBS lead itself, the above mentioned macroanatomical differences might best explain the L-R asymmetries in the change effects with stimulation seen in this study. Additionally, the networks between the left and right sides of the brain may fundamentally differ in their connectivity and functional relevance to depression (Heller et al., 1998; Herrington et al., 2010; Quraan et al., 2014; Myung et al., 2016; Poeppl et al., 2016; Li et al., 2017), which might explain our findings. The further additional mechanistic study of these tissues and networks will better contextualize the relationship between the short-term vs. long-term activity changes induced by DBS reported here linked to depression recovery.

Similarly, and contrary to expectation, was the lack of expected specificity in comparing effective to ineffective HFS within each hemisphere. A potential explanation is the obvious overlap in the location of their respective VTRs which likely affected common gray matter in and around the SCC proper, despite clear differences in the impacted white matter tracts and remote cortical regions defined by VTA-seeded tractography maps (Figure 5B). In the absence of simultaneous data from remote cortical and subcortical sites, it may be the case that local SCC changes alone cannot provide a *singular* target engagement biomarker that distinguishes effective and ineffictive HFS (or/and responders and non-responders). Rather distal electrophysiological or/and imaging recordings may be a requirement to most precisely pinpoint effective therapeutic targets with specificity. Such a conclusion is consistent with previous work demonstrating that the anatomical location of the electrode contact cannot differentiate response status (Hamani et al., 2011; Holtzheimer et al., 2017) whereas the pattern of connectivity inclusive of 4-white matter bundles can (Riva-Posse et al., 2017). Similarly, PET studies show that the local SCC decreases are consistently seen with DBS, but only remote cortical effects differentiate responders from non-responders (Mayberg et al., 2005).

Despite these caveats, differential power spectra changes in the two hemispheres with ipsilateral stimulation do suggest, as does behavioral testing, (Choi et al., 2015) that precision on the left may be more critical than on the right. Further exploration of the left theta decreases is an important next step given the consistent changes in all responders with effective left contact stimulation relative to the ineffective contacts. In addition, although not reaching significance criteria for multiple comparisons, the decrease in left theta power with left-sided stimulation was correlated with time to achieve a stable 3-week response (50% decrease on the HDRS; *p* = 0.04, *r* = 0.59), a correlation not seen with ineffective left HFS (*p* = 0.40, *r* = 0.19) or any other changes with effective stimulation.

Previous studies of SCC DBS describe 1 month changes in frontal theta cordance measured using EEG that is positively correlated with 6 month antidepressant response (Broadway et al., 2012). Furthermore, baseline ventral midline frontal theta power on EEG predicts positive antidepressant response to medication in non-TRD patients (Pizzagalli et al., 2001; Iosifescu et al., 2009; Korb et al., 2009). These converging baseline, acute and chronic theta changes at both the SCC and midline frontal cortex, point to the need for more detailed exploration of the interaction of local LFP and remote EEG oscillatory effects and continued attention to the theta signal.

### Limitations and future directions

This study had a small sample size (*n* = 14) with nearly all patients (11/14) achieving clinical response at the 6 month time point. Thus, the generalizability of our biometrics in predicting response in a broader patient population is limited.

We restricted electrophysiology signal analyses to peri-stimulation intervals due to irreversible hardware saturation from stimulation during the active DBS. The next phase of studies will examine brain oscillatory changes that are elicited during active chronic stimulation using hardware capable of simultaneous stimulation and recording off of the DBS lead (Stanslaski et al., 2012). We supposed that the left-SCC LFP power (e.g., theta to gamma bands) decreases and right-SCC LFP power (e.g., beta and gamma) decreases observed in this study provide both sufficient (but not unique) control signals in evolving closed-loop therapy and/or monitoring devices for TRD and measurable target engagement signals during extraoperative research experiments using these implantable devices.

Moreover, this study restricted analytics to frequency bands less than 50 Hz despite acquiring signals above the Nyquist sampling rate of 100 Hz and to a relative power measurement. Future studies can expand signal processing techniques to higher bandwidths and more mathematical quantifications. Perhaps high-band spectral analyses may alter the ipsilateral HFS effects (e.g., both left and right narrowband power changes) or may distinguish effective and ineffective contacts (e.g., considerable power decreases in a higher gamma band for an effective but not an ineffective DBS electrode).

Additionally, an increased sampling of the distributed depression network using invasive [e.g., electrocorticography (ECoG)] or non-invasive (e.g., high-density EEG) probes are a priority to better capture remote signaling changes induced by DBS (Smart et al., 2015), particularly if local signals cannot be shown to discriminate with more complex analyses. Differential tractography maps clearly discriminate the “effective” and “ineffective” contacts for these patients (Figure 5B), suggesting that this approach may be necessary to develop a specific electrophysiological biomarker of the optimal contact in each hemisphere, as intended.

This experiment focused on hemisphere-specific SCC effects that may guide optimal DBS lead implantation and contact selection. An alternative strategy for future research is the examination of acute bilateral stimulation (rather than only unilateral stimulation as in this work). Repeating the presented experiment but with bilateral stimulation facilitates inferences from clinically therapeutic DBS at its onset. Such investigations also enable opportunities to determine if HFS-induced power changes (e.g., left-SCC LFP theta power decreases via left effective DBS) might correlate with clinical outcomes (e.g., the time to achieve a stable therapeutic response; HDRS changes).

We anticipate and recommend that future studies can employ chronic long-term SCC LFP recordings from implanted DBS leads (Stanslaski et al., 2012) to study both acute and long-term post-operative HFS effects, including the rare chance to track near-real-time brain signal changes throughout depression symptom remission, as well as stimulation parameter optimization in novel extraoperative research. The here reported intraoperative electrophysiologic responses inform these studies to dynamically record the recovering brain amid therapeutic bilateral SCC DBS with target signals in each hemisphere that may undergo similar changes post-operatively. Future work also can combine SCC LFP recordings from the implanted device with the aforementioned extraoperative high-density EEG, intraoperative invasive cortical EEG, or ECoG via the same implantable device (Swann et al., 2018) in either setting to examine multivariate network-level activity changes [e.g., functional connectivity; (Quraan et al., 2014; Pathak et al., 2016; Fingelkurts and Fingelkurts, 2017)] and mechanistic models, such as the Wilson-Cowan network models (Goychuk and Goychuk, 2015; Cowan et al., 2016) during precise direct therapeutic stimulation of key white matter tracts. Such multimodality studies (Smart et al., 2015) may provide electrophysiological evidence that links past neuroimaging network-interaction findings in TRD patients as well as provide a more comprehensive understanding of the pathological dynamics in TRD.

## Ethics statement

This study was carried out in accordance with the Emory University IRB (Institutional Review Board) with written informed consent from all subjects. All subjects gave written informed consent in accordance with the Declaration of Helsinki. The protocol was approved by the Emory IRB.

## Author contributions

Conception and design of the study: OS, KC, PR-P, RG, AW, and HM. Care of or/and interaction with patients: PR-P, AC, and RG. Acquisition or/and analysis of data: OS, KC, PR-P, VT, AC, JE and JR. Composition of manuscript text or/and figures: all authors. Accountable of all aspects of the work: all authors.

### Conflict of interest statement

HM is a consultant with licensed intellectual property to St Jude Medical Corp (now Abbott Labs). RG has received grants from Medtronic Inc., Neuropace and MRI Interventions, honoraria from Medtronic Inc. and MRI Interventions; and is a paid consultant to St Jude Medical Corp., Medtronic Inc., Neuropace, MRI Interventions, Neuralstem and SanBio. These arrangements were approved by Emory University. The remaining authors declare that the research was conducted in the absence of any commercial or financial relationships that could be construed as a potential conflict of interest.
